# Arthroscopic Lateral Ligament Repair Through Two Portals in Chronic Ankle Instability

**DOI:** 10.2174/1874325001711010617

**Published:** 2017-07-31

**Authors:** Jorge Pablo Batista, Jorge Javier del Vecchio, Luciano Patthauer, Manuel Ocampo

**Affiliations:** 1 Centro Artroscópico Jorge Batista, 2446 Pueyrredón Avenue, 1st Floor, 1119 Buenos Aires, Argentina; 2 Favaloro Foundation, 461 Solis Street 1st floor, 1078 Buenos Aires, Argentina

**Keywords:** Ankle, Chronic instability, Arthroscopy, Reconstruction

## Abstract

**Objectives:**

Injury to the lateral ligament complex of the ankle is one of the most common sports-related injury.

Usually lateral ankle evolves with excellent clinical recovery with non surgical treatment, however, near about 30% develop a lateral chronic instability sequela.

Several open and arthroscopic surgical techniques have been described to treat this medical condition.

**Material and Methods:**

Of the 22 patients who were treated; 18 males and 4 females, and aged from 17-42 years (mean 28 years).

All patients presented a history of more than three ankle sprains in the last two years and presented positive anterior drawer and talar tilt test of the ankle in the physical examination.

We perform an anterior arthroscopy of the ankle in order to treat asociated disease and then we performed *“All inside¨* lateral ligament repair through two portals (anteromedial and anterolateral) using an anchor knotless suture.

**Results:**

Clinical outcome evaluations were performed at a mean follow up of 25 months. (R: 17-31).

Overall results has been shown by means of the American Orthopaedic Foot and Ankle Society (AOFAS). Mean AOFAS scores improved from 63 points (range 52–77) preoperatively to 90 points (range 73–100) at final follow up.

No recurrences of ankle instability were found in the cases presented.

**Conclusion:**

Several surgical procedures have been described during the last years in order to treat chronic ankle instability. *¨All inside¨* lateral ligament reconstruction presents lower local morbidity than open procedures with few complications. Moreover, it is a reproductible technique, with high clinical success rate, few complications and relatively quick return to sports activities. A high knowledge of the anatomic landmarks should be essential to avoid unwated injuries.

## INTRODUCTION

Injury to the lateral ligament complex of the ankle is one of the most common sports-related injury and the incidence of ankle sprains is reported to be as high as 45% in basketball players and up to 31% in soccer players [1, 2]. Usually, lateral ankle sprain evolves with excellent clinical recovery with non surgical treatment; however, near about 30% develop a lateral chronic instability sequela. The associated pain and instability often disable the patients to participate in sports activities. Typical symptoms include pain during or after activity, recurrent swelling, “giving way” feeling and weakness. Repetitive ankle sprains and persistent symptoms after injury have been termed chronic ankle instability (CAI) [3, 4]. Two contributing factors to CAI are mechanical ankle instability and functional ankle instability [5-7]. There are, however, numerous insufficiencies that lead to each type of instability. Mechanical insufficiencies include pathologic laxity, impaired arthrokinematics, and synovial and degenerative changes [8]. Functional insufficiencies include impaired proprioception, altered neuromuscular control, strength deficits, and diminished postural control [6]. Although mechanical and functional instability may occur in isolation, researchers have shown that combinations of the two most likely contribute to CAI [6-8].

Several surgical techniques have been described for the treatment of chronic lateral ankle instability. They can be divided into anatomic and nonanatomic reconstruction procedure. Arthroscopy is an alternative to treat these patients, but it is traditionally known that this procedure is only used in diagnostic purposes and the treatment of associated intra-articular injuries. Historically, the stabilization itself was openly performed because standard anterior ankle arthroscopy provided only partial visualization of the anterior talofibular ligament from above and the calcaneofibular ligament attachments cannot be seen at all [3, 9]. Although several publications with good outcomes have been reported in the last fourty years described by open procedures, there is increasing interest in the implementation of arthroscopy stabilize the ankle joint [10-14]. The pain can be originated by osteochondral lesions, soft tissue or bone impingement syndromes and others pathologies that should be treated before the ligament repair [13].

The arthroscopic anatomic ligament repair technique for lateral collateral chronic instability does not require the creation of a third accesory portal and it isperformed through the two classic anteromedial and anterolateral portals described by V. Dijk [15]. We described a safe and reproducible “*All inside”* arthroscopic procedure in 22 patients with symptomatic mild and moderate chronic ankle instability to repair the anterior talofibular ligament (ATFL) or perform an augmentation technique in case of rupture of the ATFL and/or calcaneofibular ligament (CFL) maintaining all the advantages of an arthroscopic approach. Arthroscopic examination of the ankle joint has to be preceded in all cases of inside ligament repair in patients with chronic lateral instability.

## MATERIALS AND METHODS

In this study, 22 (18 males and 4 females) patients were prospectively evaluated undergoing surgery between February 2012 and January 2014. The mean aged was 28 years, with a range of 17-42 years.

Clinical outcome evaluations were performed at a mean follow up of 25 months (R: 17-31).

All patients practiced different types of sports and presented a history of more than three ankle sprains in the last two years and presented positive anterior drawer and talar tilt test of the ankle in the physical examination. Magnetic resonance imaging (MRI) examinations were performed in all patients to investigate associated intra-articular lesions and to evaluate the integrity of the lateral ligaments.

We perform arthroscopic examination of the ankle in order to treat associated conditions and then we performed*“All inside¨* lateral ligament repair though two portals (anteromedial and anterolateral) using an anchor knotless suture.The main author (JPB) performed all surgical procedures.

Overall results have been shown by means of the American Orthopaedic Foot and Ankle Society (AOFAS) score. The participation consent form was correctly informed and signed by all patients.

### Surgical Technique

**Fig. (2):**
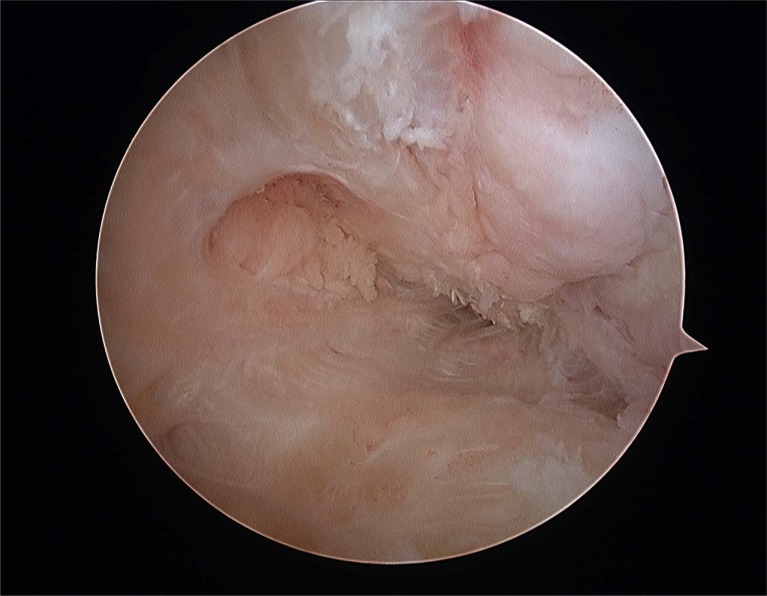
Lateral gutter.

**Fig. (3):**
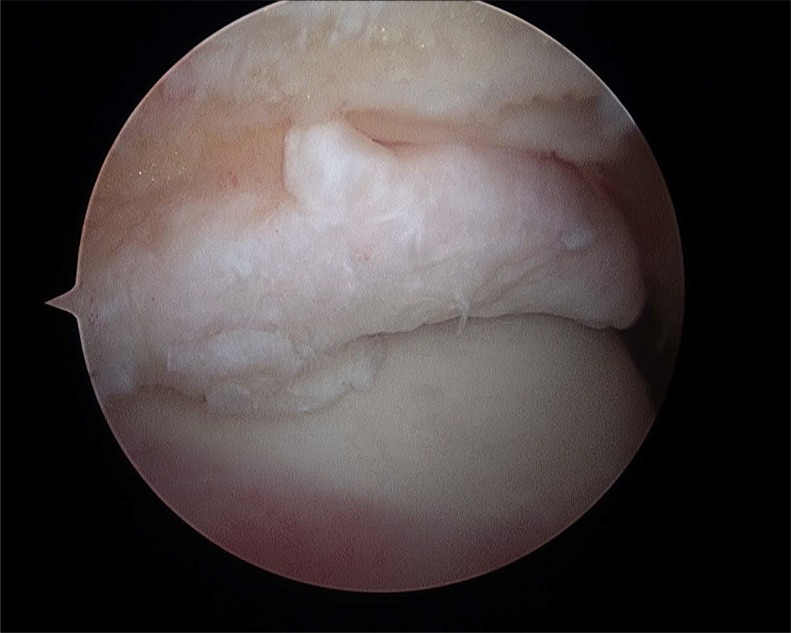
Tibial exostosis.

The patient was placed in supine position, both the hip and the knee were extended with the ankle on the tip of the table to allow flexo-extension movement during the surgery. Only two arthroscopic portals are needed: the classic, well knowledge anteromedial and anterolateral portal described by Prof. V.Dijk [15] Fig. (**1**). We identify and perform landmarks on the anterior joint line, which is easily palpated by moving the joint through plantar and dorsiflexion; the anterior tibialis tendon (that mobilized lateral in dorsiflexion); the peroneus tertius tendon which is present in 90% of cases; the lateral malleolus; and the superficial peroneal nerve [16-18]. Clinical examination maneuvers to identify that the nerve was performed systematically, the nerve moves with ankle motion, and its subcutaneous course becomes evident with inversion of the ankle and plantarflexion of the fourth toe [19, 20]. Distraction of the ankle was not used during this arthroscopic procedure routinely. A 4-mm 30° arthroscope is introduced through anteromedial portal close to the anterior tibial tendon. The ankle is positioned in maximum dorsiflexion to obtain the optimal view of the lateral gutter (Fig. **2**). In this position, anterolateral portal is made by transillumination taking care of the superficial peroneal nerve. We explored the anterior tibio-talar joint and removed sinovial processes, tibial spurs, osteophytes and talar beaks (Fig. **3**). We suggest blunt disection of the subcutaneous tissue in order to improve the working area on the lateral gutter. Dissection with a shaver starts on the lateral side with debridement and visualization of the anteroinferior tibiofibular ligament (AITFL). The dissection continues distally by releasing the superior part of the ATFL from the malleolus to the talus. After this stage, the dissection continued outside the joint on the lateral side of the ATFL. After a full arthroscopic exploration, the lateral collateral ligaments were repaired under direct arthroscopic view.

**Fig. (5):**
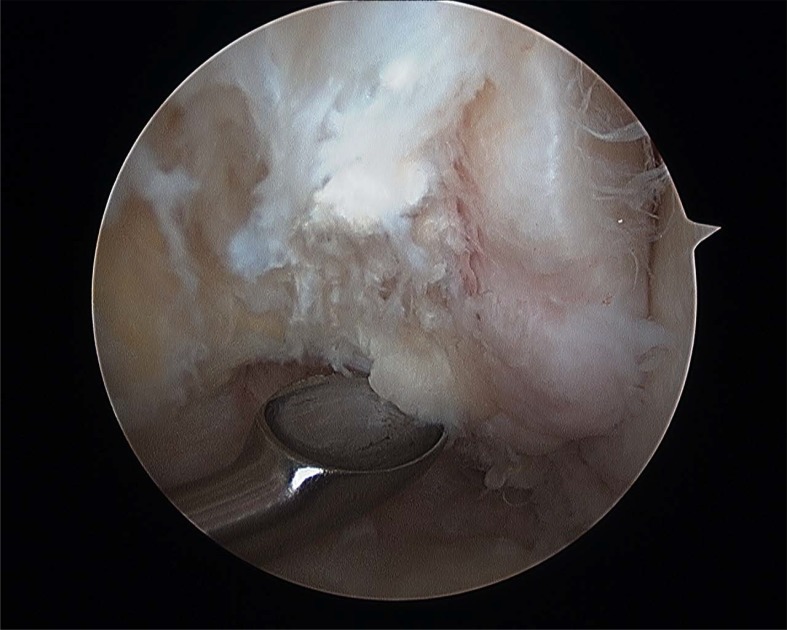
Fibular footprint ATFL.

Prior to reattach the ligament, it should be defined if the anterior talofibular ligament present a parcial or complete lesion and if the calcaneofibular ligament (CFL) is broken (Figs. **4A**- **B**). The footprint for the fibular attachment of the lateral collateral ligaments is debrided with a shaver or a curette introduced through the anterolateral portal. [3, 10, 19, 21] (Fig. **5**) A suture passer (First Pass-Parcus), a 2:0 or 0 nonabsorbable suture HIFI (ConMed Linvatec FL), and a 4,5mm knotless anchor (Poplock 4.5 mm, ConMed Linvatec, FL) were used for ligament repair (Figs. **6A**-**B**). Perform the hole on the footprint in the distal tip of the fibula through the iniciator of the anchor by impactation. The drill was directed from anterior to posterior, and parallel to the plantar plane as well as the plane of the lateral gutter (Figs. **7A**-**B**-**C**). The First Pass suture passer is introduced through the anterolateral portal, and under direct arthroscopic visualization, the remanent ATFL or the superior retinaculum is penetrated from lateral to medial. The suture is pulled back with the first pass gripper through the anterolateral portal (Figs. **8A**-**B**-**C**). Pull back the suture to know if there is a good capture of the remanent tissue. The limbs of the suture are passed through the loop in each side of the knotless anchor (Fig. **9**). The sutures are passed through the knotless anchor (Figs. **10A**-**B**) carefully that the tension of the suture can be modulated before introducing the anchor. Once the anchor is introduced, the tension of suture can not be controlled (Figs. **11A**-**B**). (Figs. **12A**-**B**-**C**) A compressive bandage and a walking boot keeping the ankle in 90 degrees is indicated in all patients and maintained for 4 weeks. Crutches are used for two weeks. During the first week no weight bearing is indicated. Seven days after surgery the patients showed partial weight bearing, after this time they are allowed to deambulate with the walking boot.

## RESULTS

Clinical outcome evaluations were performed at a mean follow up of 25 months (R: 17-31).

All patients presented a stable ankle without pain or instability after the surgery. No patient with recurrence of lateral ankle instability was detected. Mean AOFAS scores improved from 63 points (range 52–77) preoperatively to 90 points (range 73–100) at the final follow up. No complications were found, neither infection nor wound complications or neurologic Turing follow-up. Nevertheless, two patients complained pain in the anteromedial portal after the surgery, which improved during follow-up though deep massage during the rehabilitation period.

Tenodesis procedures, most often using the peroneus brevis tendon, are commonly described for stabilization of the lateral ankle [32]. These procedures have been shown to provide stability of the lateral ankle with satisfactory shortterm results, but the long-term results have deteriorated with time [27]. These procedures involve extensive surgical exposure, dificult techniques, and prolonged immobilization and are associated with weakness of the peroneal tendons with reduction in the range of motion, including loss of inversion and associated loss of eversion strength.

VanDijk *et al* [32] reported 21 patients with chondral injuries, six with loose bodies, and 19 with traumatic synovitis in 20 patients who had arthroscopy before primary repair of ruptured ligaments.

Regarding arthroscopy, studies have shown that converting to an arthroscopic technique can be demanding, with a long learning curve, a great knowledge of the arthroscopic anatomy and an increase in surgical time; however, the advantages of preserving the soft-tissue envelope and the improved visualization outweigh these issues [9-11, 15-18, 39].

The arthroscopic ligament repair through the two classic portals is technically demanding, but the most difficult step in the procedure is the lateral ankle endoscopic recognizing of the ligament structures and bone landmarks and dissection of its, and we recommend cadaveric training prior to perform the technique. These techniques, which are technically demanding, have shown early promise in Level IV studies with short-term follow-up.

The use of a knotless suture anchor technique showed advantages over traditional suture anchors. The possibility of a prominent anchor or suture knot were avoided with the new knotless anchors.

This study was limited by the fact that a control group was not included to compare the outcome with the arthroscopic ligament repair procedure described.

As complications, Ferkel *et al*. presented a complication rate of 9.0% associated with ankle arthroscopy. Neurological injuries accounted for the largest portion of these complications [33] V. Dijk demostrated a 1.9% of neurological complications and it compares favorably to the average of 3.7% reported in the literature [35-39]. We did not report any neurologic complications in our first 22 cases.

We presented 2 intraoperative complications in which the anchor was broken during the impactation Fig. (**13**).

Soft-tissue complications like infection, wound dehiscence, and hiperesthesia in the sural nerve area were reported significantly more in open ankle and foot surgery when compared with other orthopaedic procedures. In addition, we believe that the use of the arthroscopy lonely technique might reduce these higher rates of complications. We found that preliminay results are promising but further investigation and reporting of results are required before the described technique can be adopted as a standard procedure. Although we presented a short number of cases and the study design did not allow us to provide a comparative analysis with other established procedures, the arthroscopic lateral ligament repair is a reproductible technique, with very high succefull rates, low complications and relatively early return to sports activities.

## CONCLUSION

The goals of anatomic repair are to restore the anatomy and joint mechanics and to maintain ankle and subtalar motion. Anatomic procedures, such as the modified Brostrom and others show force patterns during loading similar to those seen in intact ankles [22, 23]. The modified Brostrom procedure, as described by Gould, produces stability of the lateral ankle, while preserving range of motion [24]. Reports on the Brostrom procedure have demonstrated results equal to or superior to those of the reconstruction procedures, with less postoperative pain, less instability, and no associated loss of inversion or eversion strength [25-28]. Liu and Baker [29] studied the static restraints of various operative procedures in 40 cadaver ankles. The five groups included 1) intact ATF and CF ligaments; 2) incised ATF and CF; 3) Chrisman-Snook reconstruction; 4) Watson-Jones reconstruction; and 5) modified Brostrom repair. The results of the mechanical anterior drawer and inversion stress were recorded. Their conclusions were that all procederes decreased the anterior drawer and talar tilt. There was no significant difference between the Watson-Jones [30] and Chrisman-Snook [31] procedures, but the modified Brostrom [25] had the minor anteroposterior displacement and talar tilt at all different forces tested.

## Figures and Tables

**Fig. (1):**
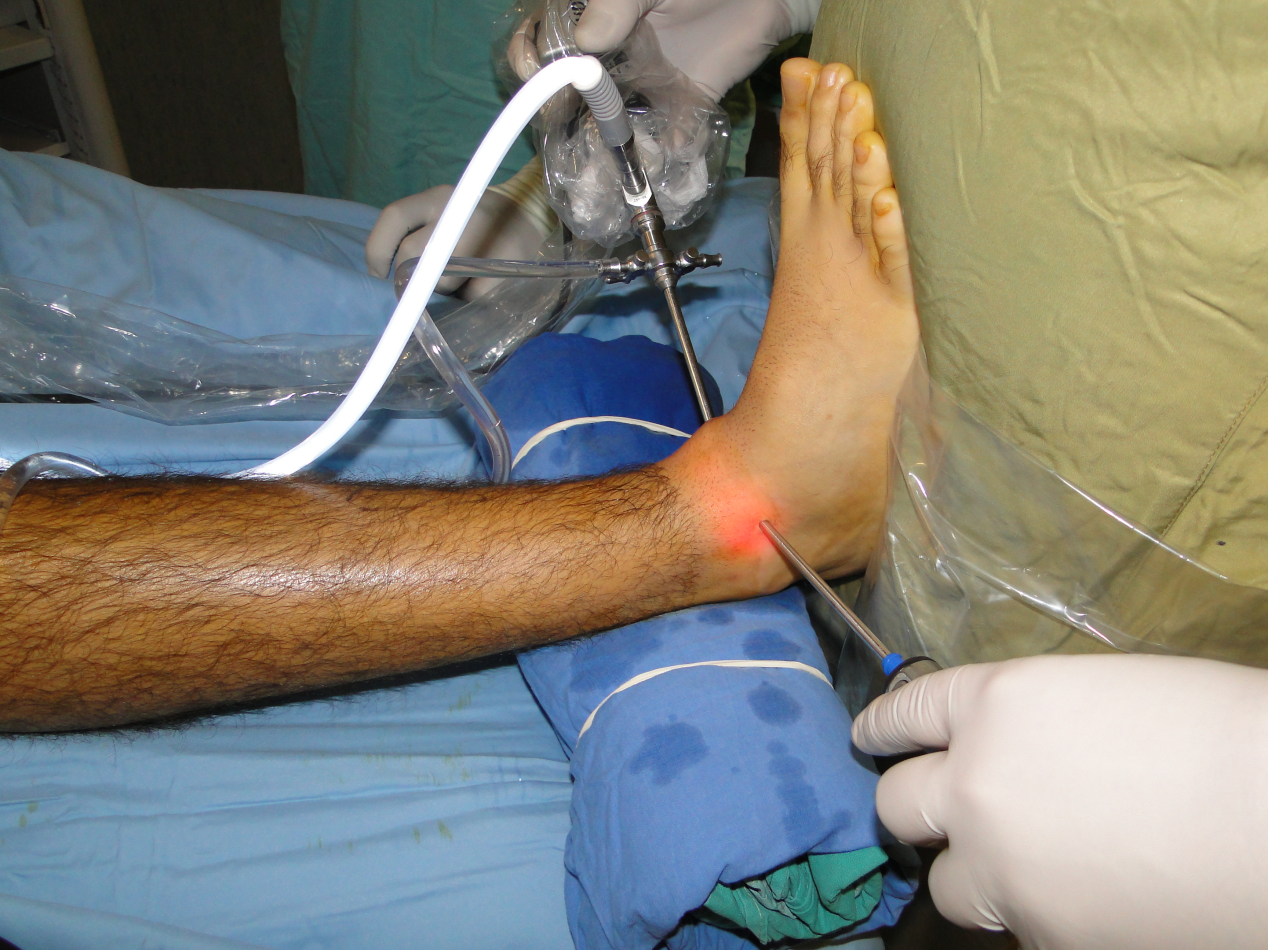
Arthroscopic approach in dorsiflexion (Anteromedial and anterolateral portals).

**Fig. (4A):**
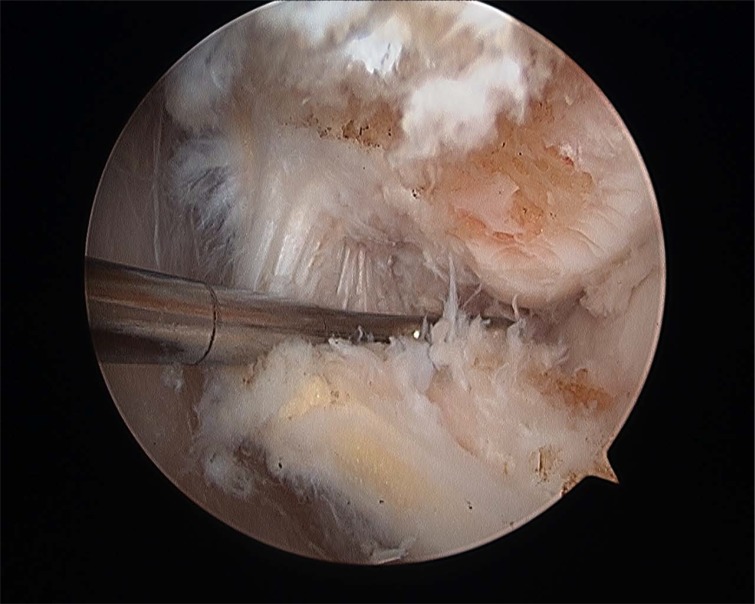
Anterior talofibular partial rupture.

**Fig. (4B):**
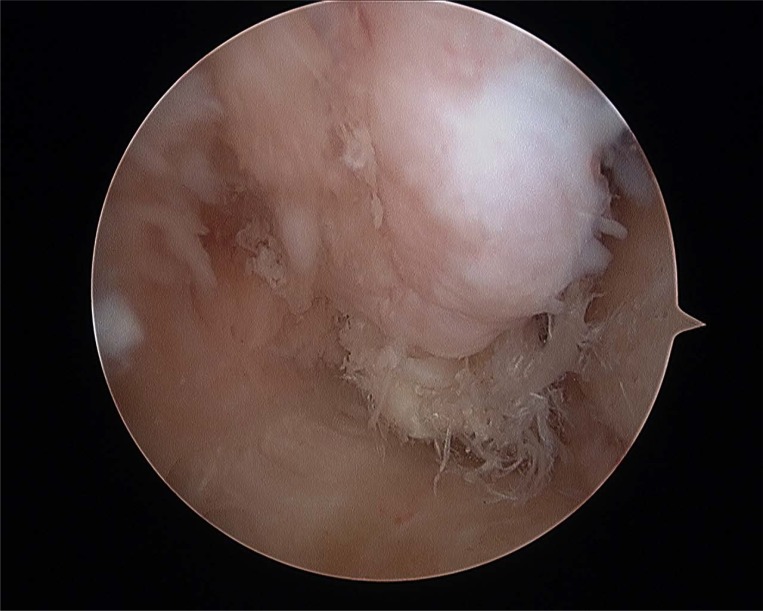
Complete anterior talofibular rupture.

**Fig. (6A):**
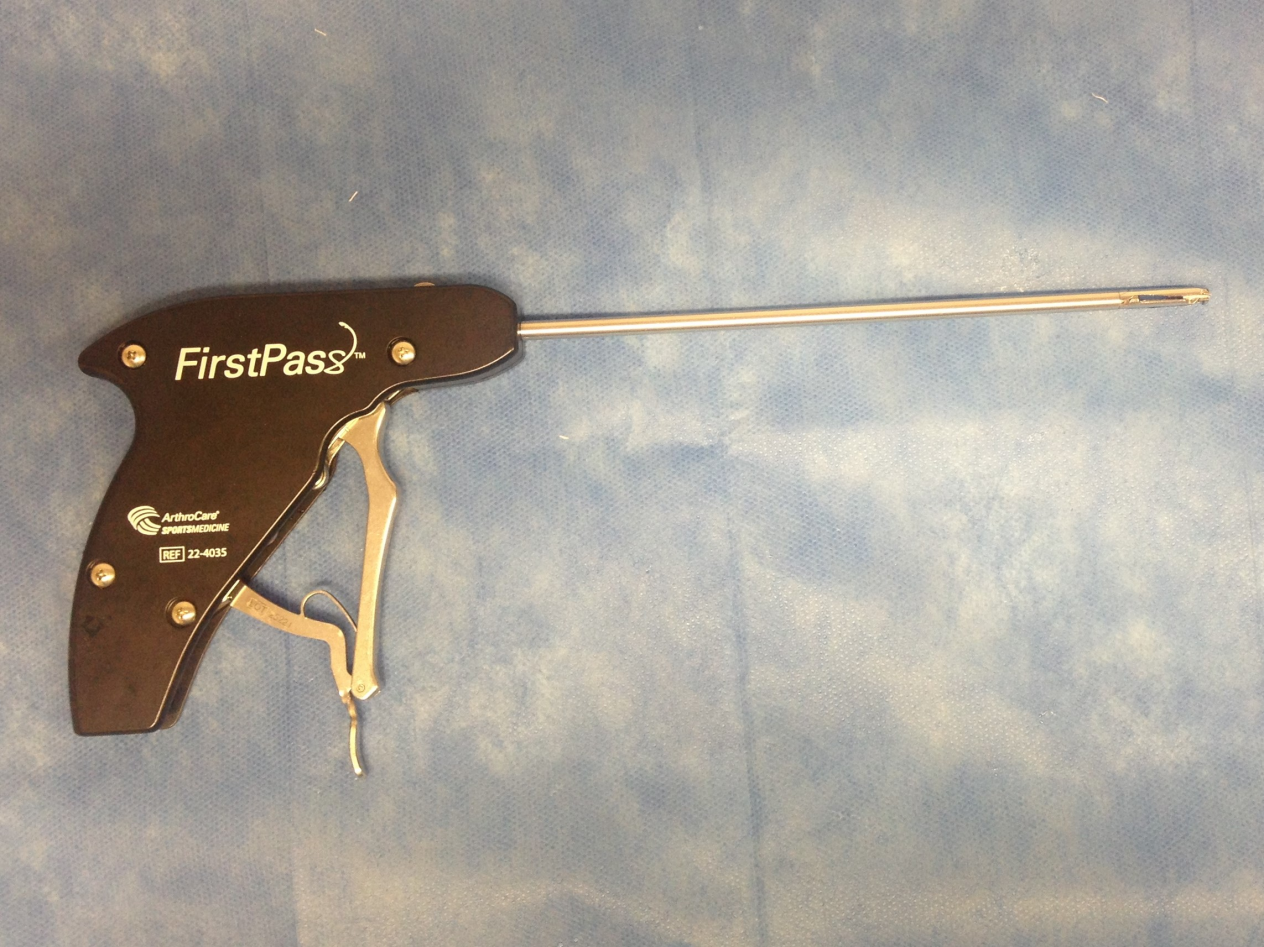
First Pass Suture Passer.

**Fig. (6B):**
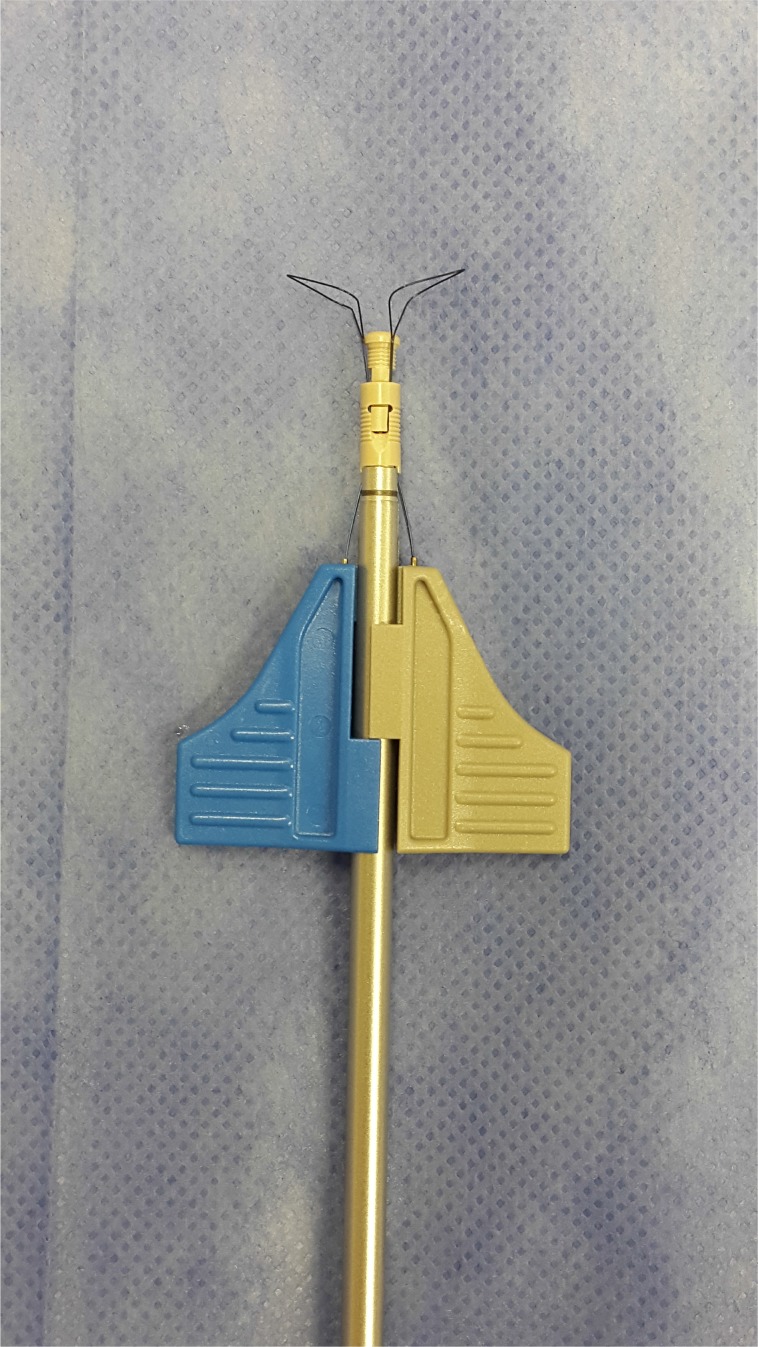
Anchor knotless suture 4, 5mm.

**Fig. (7A):**
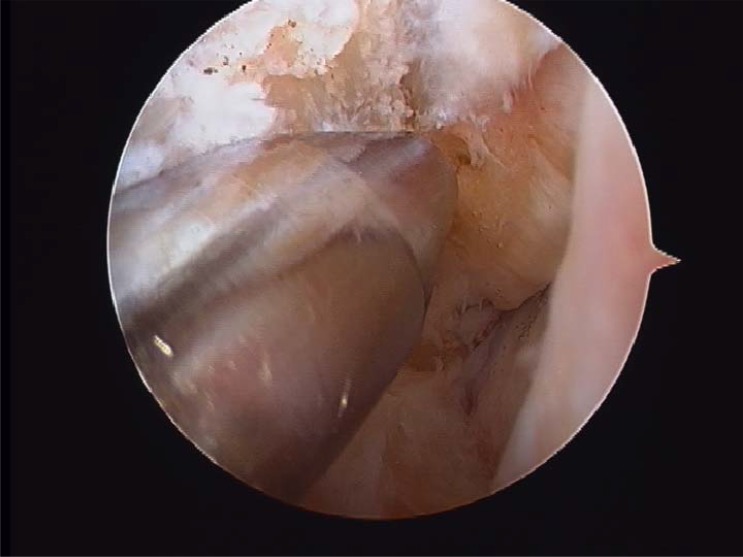


**Fig. (7B):**
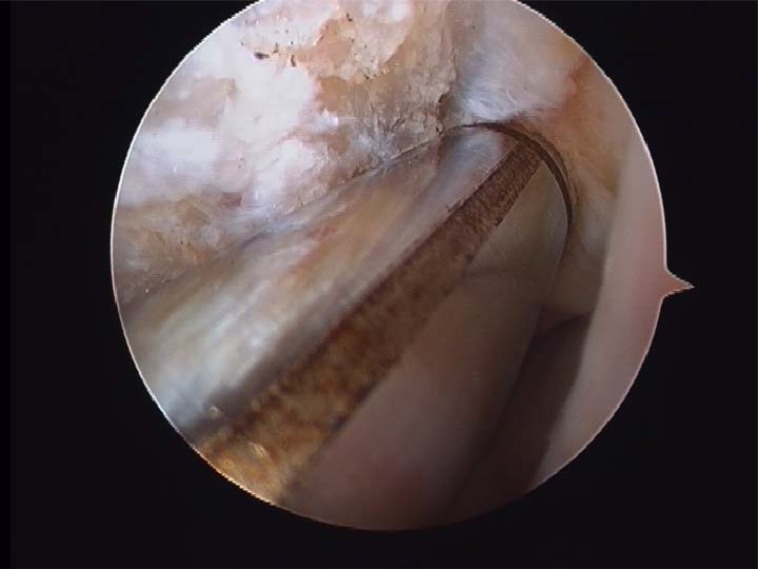


**Fig. (7C):**
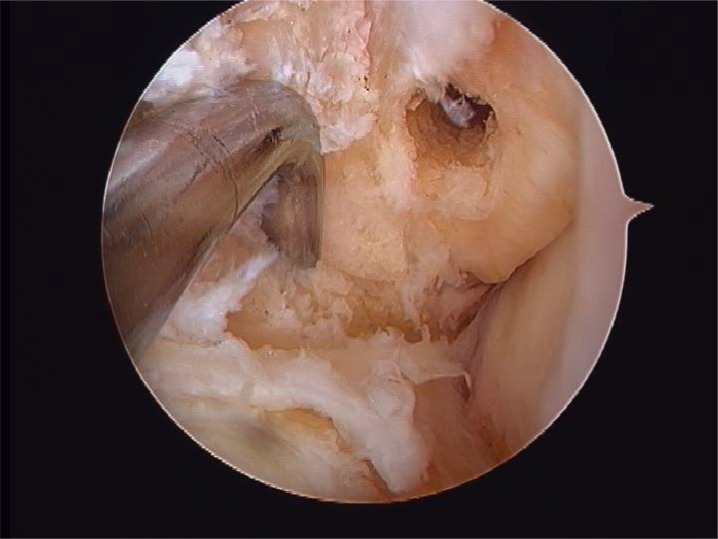
(Sequence Pictures) Performing the hole in fibular´s footprints.

**Fig. (8A):**
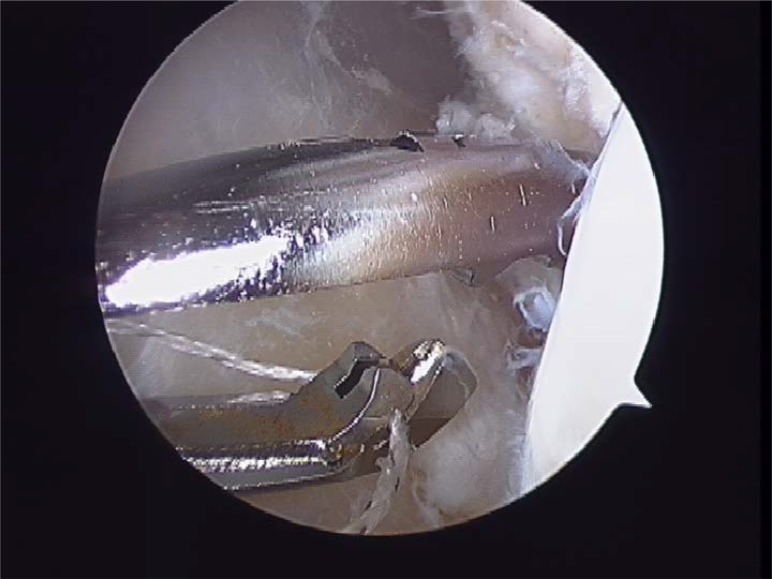
(Sequence Pictures) Grasping the ligament with a HIFI suture is the first step for the repair. (A).

**Fig. (8B):**
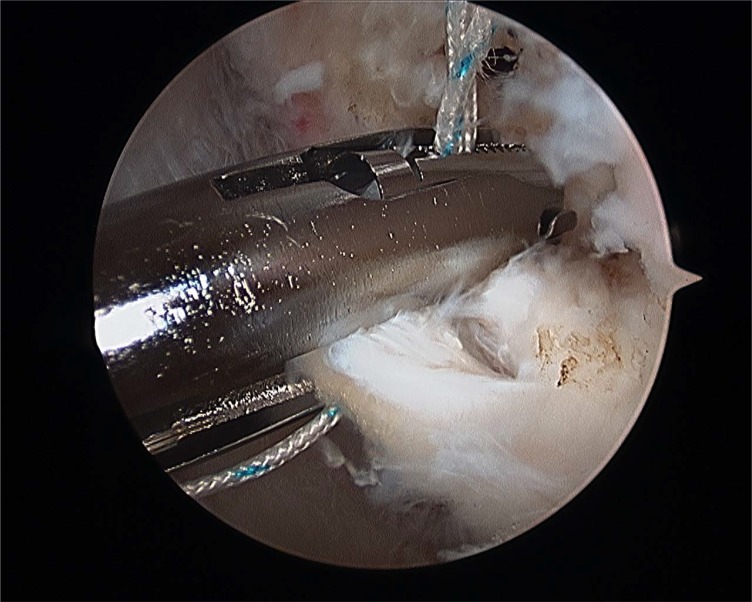
The First Pass suture passer is introduced through the anterolateral portal and the ligament is penetrated from lateral to medial (B).

**Fig. (8C):**
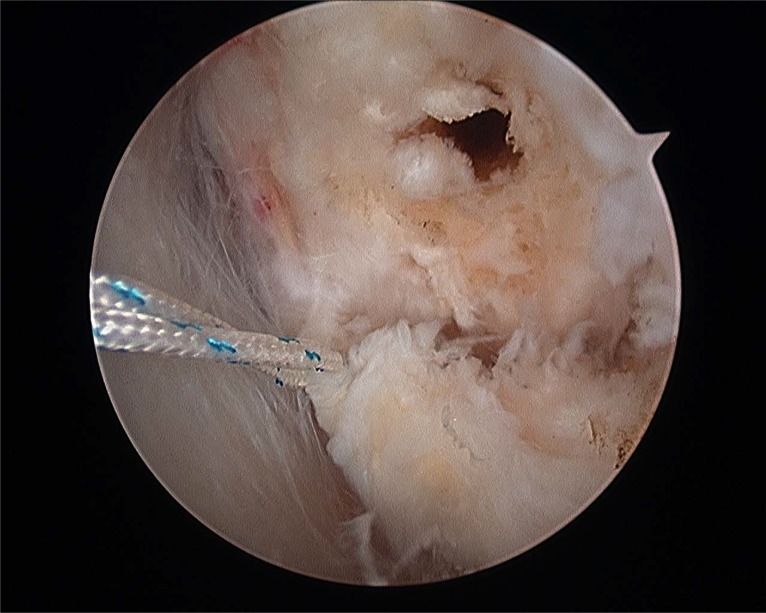
Pull back the suture to know if there is a good capture of the remanent tissue (C).

**Fig. (9A):**
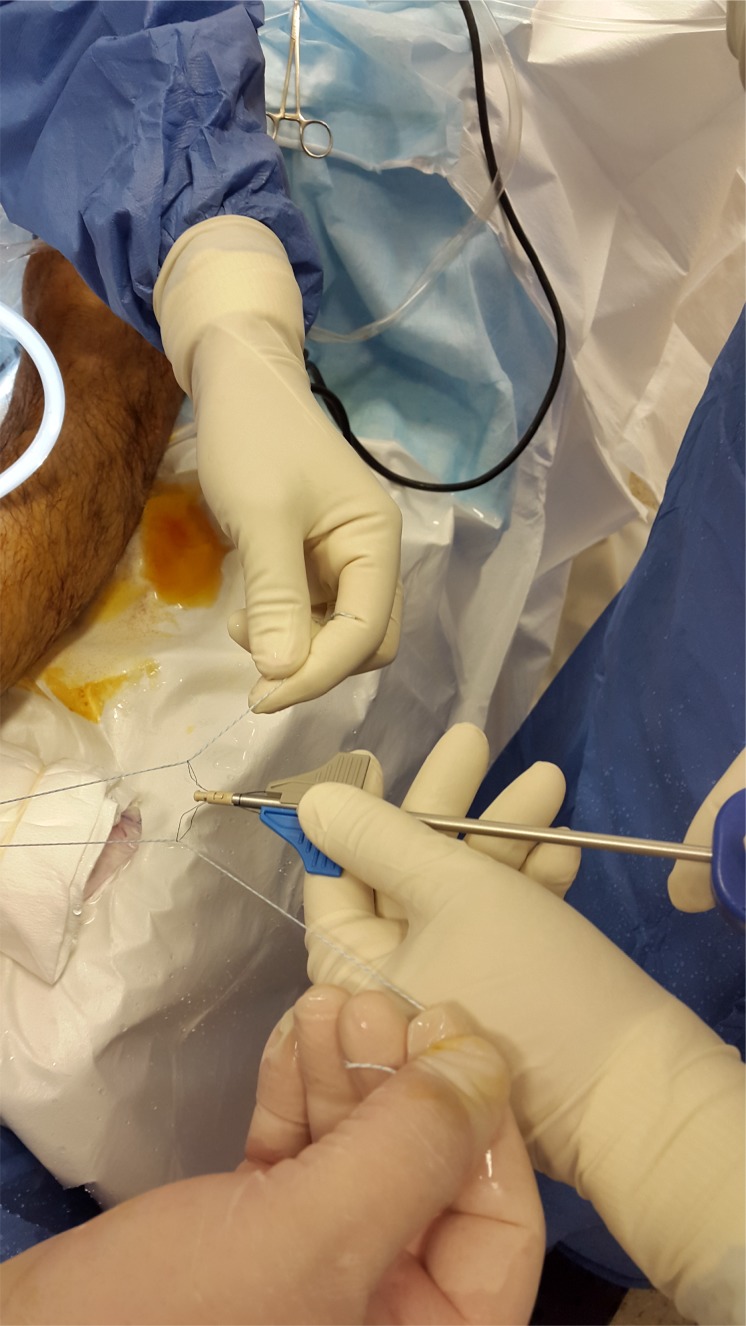


**Fig. (9B):**
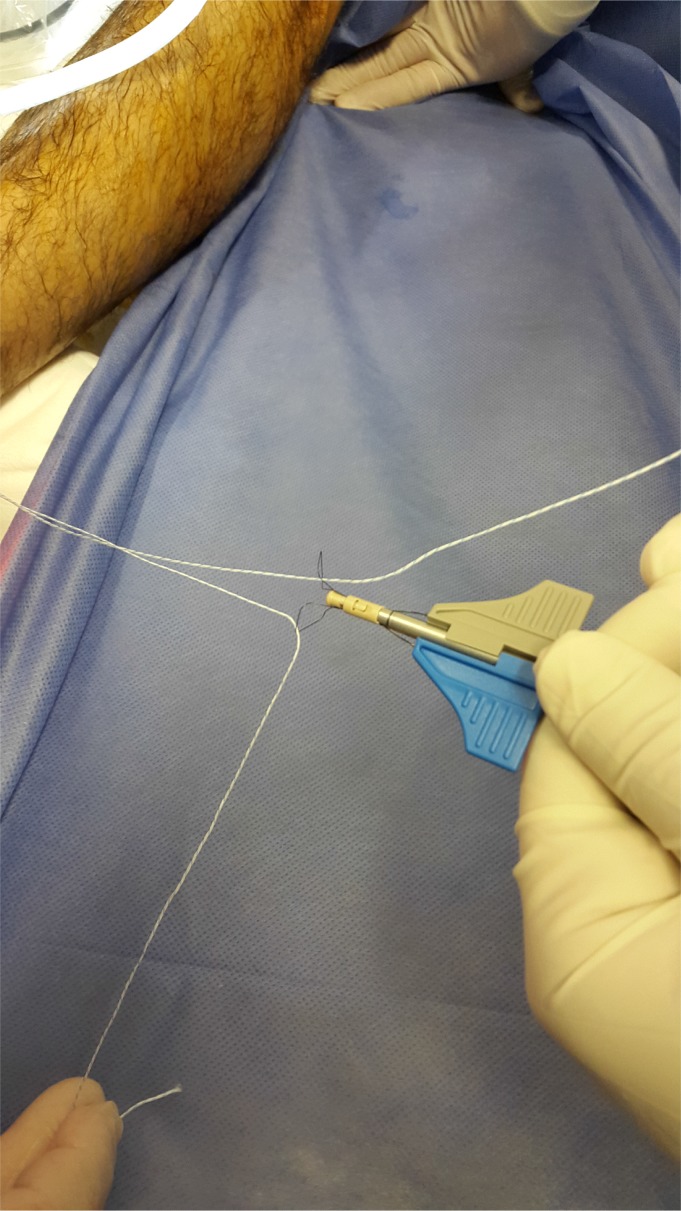
Introducing the sutures into de anchor knotless loop.

**Fig. (10A):**
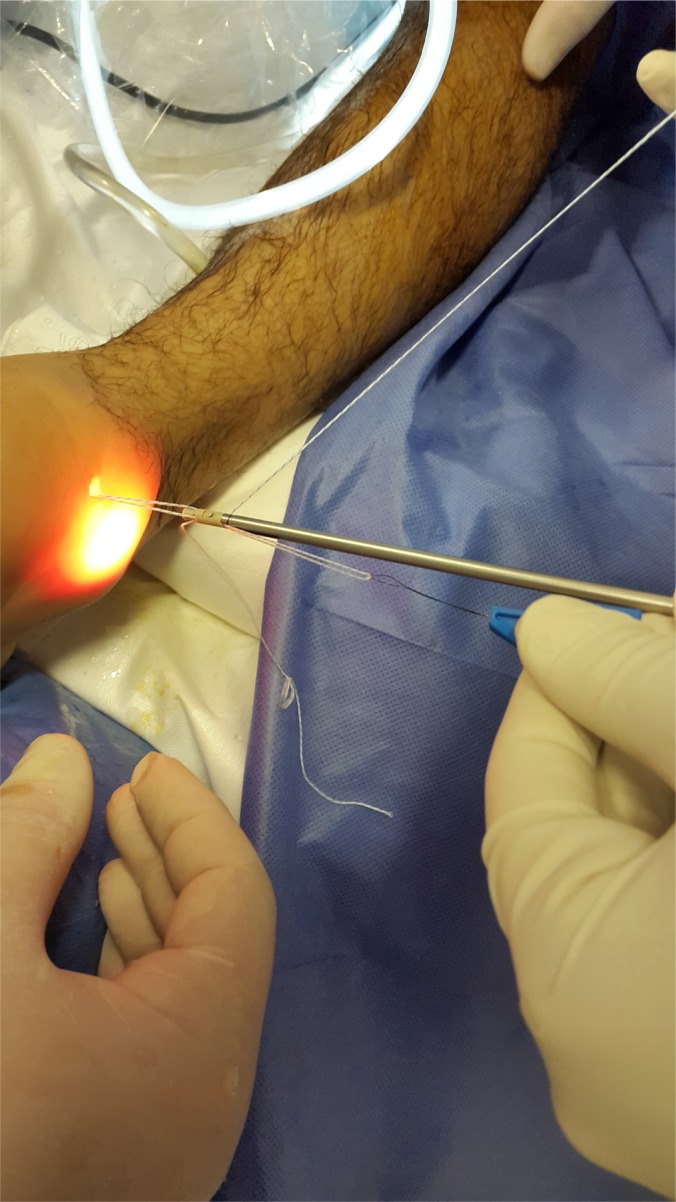


**Fig. (10B):**
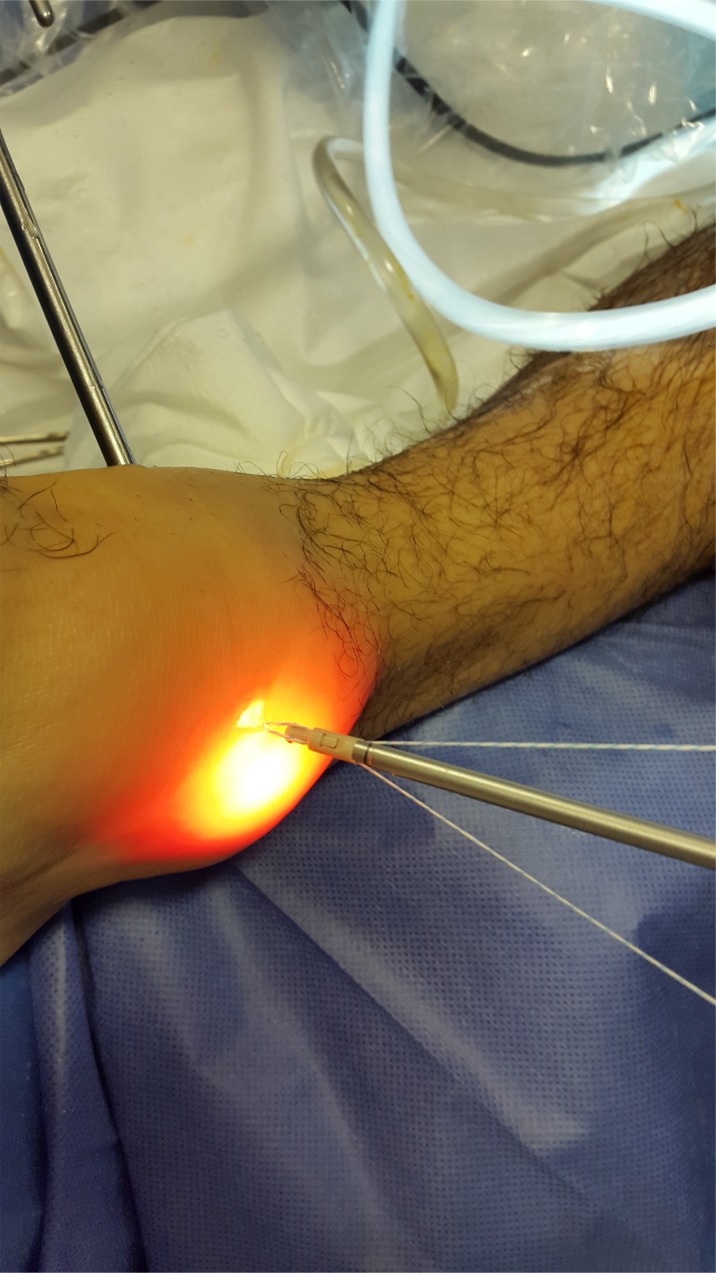
Introducing the anchor.

**Fig. (11A):**
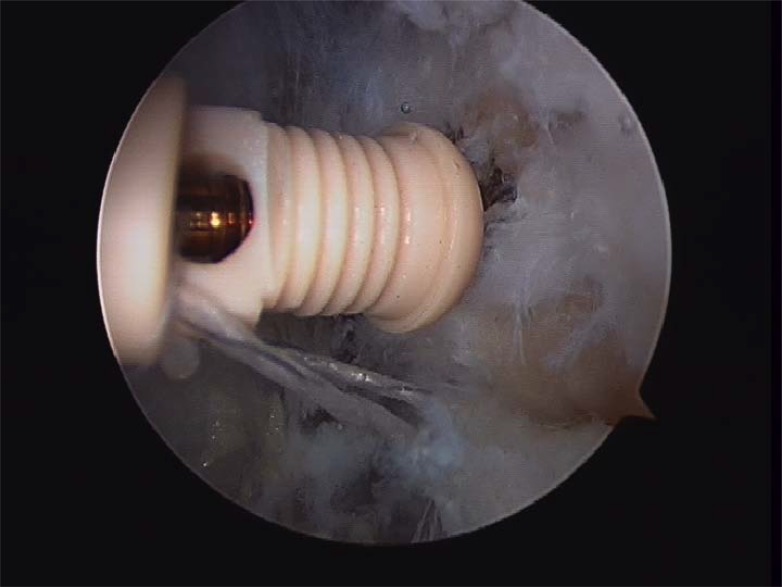
Impacting the anchor and performing tension through the suture and remanent tissue.

**Fig. (11B):**
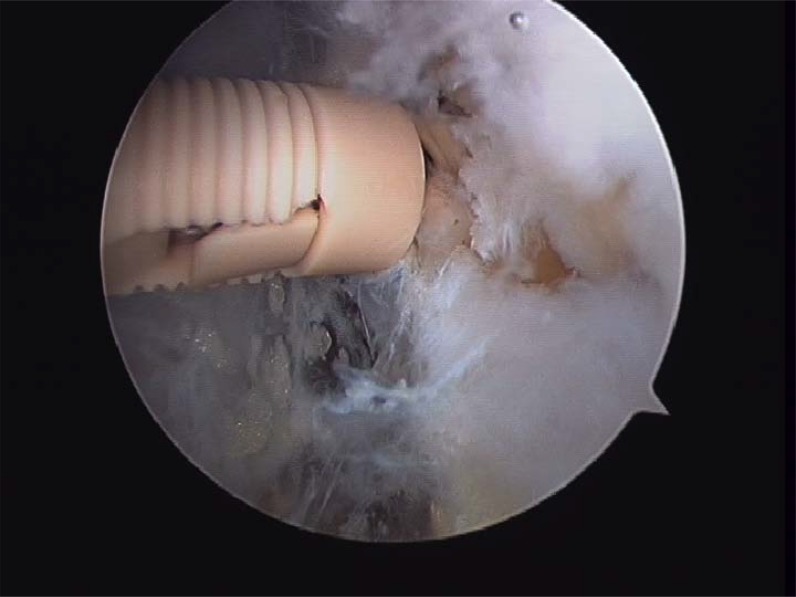
Impacting the anchor and performing tension through the suture and remanent tissue.

**Fig. (12A):**
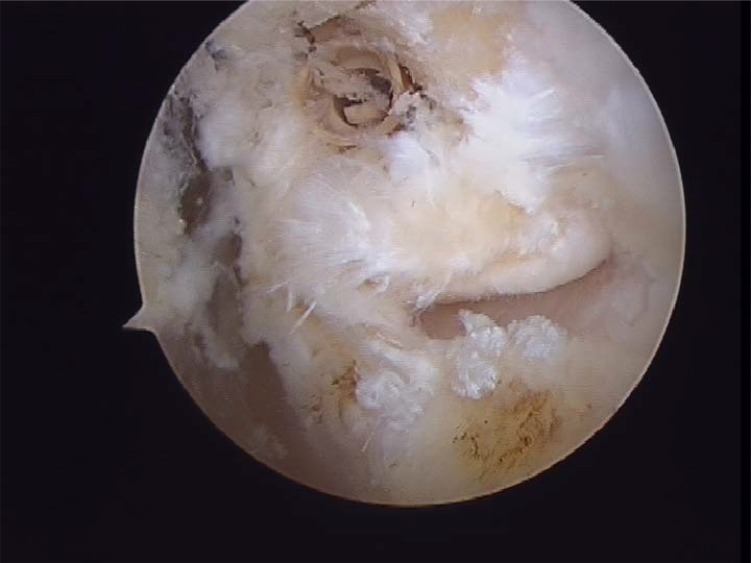
Final ligament repair . The ligament is attached on its footprints and can be seen from anterolateral portal.

**Fig. (12B):**
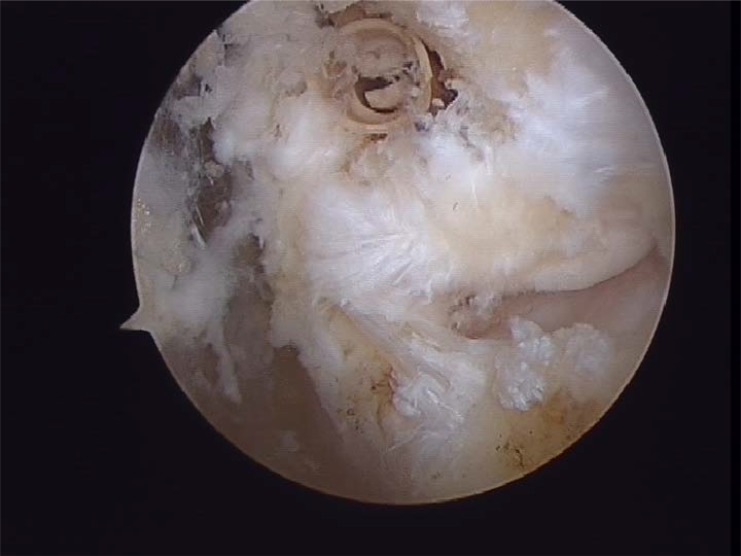
Final ligament repair . The ligament is attached on its footprints and can be seen from anterolateral portal.

**Fig. (12C):**
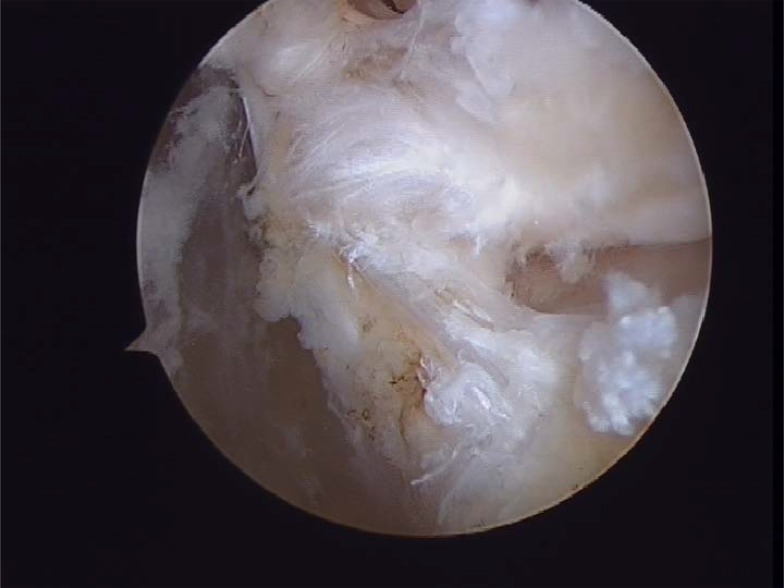
Final ligament repair . The ligament is attached on its footprints and can be seen from anterolateral portal.

**Fig. (13):**
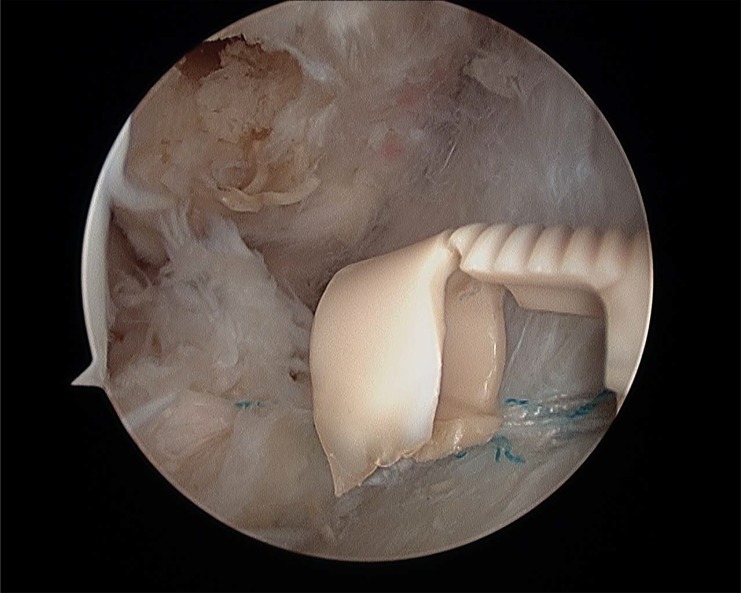
Anchor rupture during the impactation.
